# Study of the Influence of the Irradiation Flux Density on the Formation of a Defect Structure in AlN in the Case of the Effect of Overlapping of the Heavy Ion Motion Trajectories in the Near-Surface Layer

**DOI:** 10.3390/ma16155225

**Published:** 2023-07-25

**Authors:** Yeugeniy V. Bikhert, Artem L. Kozlovskiy, Anatoli I. Popov, Maxim V. Zdorovets

**Affiliations:** 1 Engineering Profile Laboratory, L.N. Gumilyov Eurasian National University, Satpaev Str. 5, Astana 010008, Kazakhstan; kozlovskiy.a@inp.kz (A.L.K.);; 2 Laboratory of Solid State Physics, The Institute of Nuclear Physics, Almaty 050032, Kazakhstan; 3 Institute of Geology and Oil and Gas Business, Satbayev University, Almaty 050032, Kazakhstan; 4 Institute of Solid State Physics, University of Latvia, 8 Kengaraga Str., LV-1063 Riga, Latvia; popov@latnet.lv

**Keywords:** AlN ceramics, radiation defects, heavy ion irradiation, optical properties, deformation distortions

## Abstract

The aim of this paper is to test the previously stated hypothesis and several experimental facts about the effect of the ion flux or ion beam current under irradiation with heavy ions on the radiation damage formation in the ceramic near-surface layer and their concentration. The hypothesis is that, when considering the possibilities of using ion irradiation (usually with heavy ions) for radiation damage simulation at a given depth, comparable to neutron irradiation, it is necessary to consider the rate factor for the set of atomic displacements and their accumulation. Using the methods of X-ray diffraction analysis, Raman and UV–Vis spectroscopy, alongside photoluminescence, the mechanisms of defect formation in the damaged layer were studied by varying the current of the Xe^23+^ ion beam with an energy of 230 MeV. As a result of the experimental data obtained, it was found that, with the ion beam current elevation upon the irradiation of nitride ceramics (AlN) with heavy Xe^23+^ ions, structural changes have a pronounced dependence on the damage accumulation rate. At the same time, the variation of the ion beam current affects the main mechanisms of defect formation in the near-surface layer. It has been found that at high values of flux ions, the dominant mechanism in damage to the surface layer is the mechanism of the formation of vacancy defects associated with the replacement of nitrogen atoms by oxygen atoms, as well as the formation of *ON–VAl* complexes.

## 1. Introduction

In modern structural material science, experimental works related to the study of the resistance of materials to radiation damage caused by various types of radiation occupy a special place. Interest in such studies is primarily due to the need to obtain new data on the radiation resistance of materials planned to be used as materials for nuclear energy, as well as to study the dynamics of their properties under the influence of radiation [1,2,3]. Moreover, over the past few years, studies related to the radiation resistance of various types of ceramics based on oxide or nitride compounds, which have several characteristics that allow them to be used in extreme conditions associated with high temperatures and high-dose radiation, have been of great interest [4,5]. One of the key properties of ceramic materials that allow them to be used as structural materials is their resistance to the accumulation of radiation damage and subsequent phase-structural transformations associated with the accumulation effects of structural distortions and deformation stresses in the damaged layer [6,7]. Unlike metal alloys and steels, in which the processes of martensitic transformations can be initiated during high-dose irradiation, which result into a sharp decrease in the strength properties of materials, such processes are not observed in ceramics in view of their structural features. However, in several cases, so-called latent tracks (structural disordering areas) were found in ceramic materials, the formation of which leads to the destabilization of the damaged layer and its disordering with subsequent amorphization [8,9,10]. At the same time, the formation of latent tracks in ceramic materials, as shown in several papers [9,10,11,12], has a pronounced dependence on the energy and type of incident ions, the variation of which leads to different rates of formation and accumulation of radiation damage in the form of point and vacancy defects, as well as changes in electronic density due to the differences in ionization losses. However, as is known, the radiation damage accumulation rate can be influenced not only by the energy of the incident ions and their type, but also by their flux density, i.e., the number of ions interacting with materials at a time [13,14]. This issue, in most cases, is not considered in view of large radiation fluences (as a rule, the accumulation of radiation damage and major structural changes is observed at fluences above 10^12^ ion/cm^2^), as well as a long exposure time, which in the case of large doses can be several tens of hours [15,16].

The purpose of this work is to assess the effect of ion flux density during heavy ion irradiation on the formation of a defective structure in the near-surface layer of ceramics at the same irradiation flux. As is known, the use of heavy ion irradiation of structural materials can generally be used to simulate radiation damage in material caused by neutron exposure in nuclear reactors. However, with a more in-depth study, several factors must be considered for the use of heavy ions for such simulation conditions.

First, the depth of the damaged layer of structural materials under irradiation with heavy ions is much less than under neutron exposure, in view of the fact that neutrons have a greater penetrating power than ions [17,18]. In this regard, when interpreting the data obtained, it is necessary to make a clarification that irradiation with heavy ions makes it possible to simulate radiation damage at a certain depth or in a near-surface layer with a thickness of no more than 10–30 μm.

Secondly, when using heavy ion irradiation for comparison with neutron exposure, it is necessary to use commensurate values characterizing radiation damage, such as atomic displacements (dpa) [19,20]. At the same time, in the case of high-dose neutron irradiation of structural materials, the observed structural changes are typical at displacements from 0.1 to 30–50 dpa, and in some cases even higher values > 100 dpa. In this case, when irradiated with high-energy heavy ions (with an energy of more than 1 MeV/nucleon), the achievement of similar dpa values occurs only in the case of fluences above 10^15^ ion/cm^2^. At such irradiation fluences, a significant role in the radiation damage accumulation processes is played by the effects of possible partial sputtering or embrittlement of the surface during prolonged irradiation, as well as the effects of overlapping of damaged areas that appear along the ion motion trajectory in the material of the near-surface layer [21,22]. It is also necessary to consider the time frame of exposure. Thus, for neutron irradiation, the achievement of large displacements takes several years, sometimes tens, while during irradiation with heavy ions, such values are achieved for several days or weeks, depending on the flux density of incident particles. In this regard, such a large difference in the time of accumulation of radiation damage must also be considered in a comparative analysis of damage and interpretation of the data obtained.

Previously, it was shown in [23] that, under irradiation with low-energy ions, the variation in the flux value leads to differences in the main mechanisms of defect formation, which must be considered when further comparing the possibilities of radiation damage simulation. In the conclusion of this study, the authors report that when using ion irradiation to simulate the neutron impact on nuclear materials, one should consider the effects of cascade mixing that occur during high-dose irradiation, as well as the high radiation density in the case of ion irradiation. Moreover, when using ion irradiation to simulate radiation damage in the event of accelerated irradiation in the structure of the damaged layer of material, negative effects may occur due to the rate of formation of defects and their subsequent evolution. At the same time, if these effects can be partially reduced for steels and alloys by the presence in the structure of impurities or many boundary and dislocation defects, as well as various nanostructured inclusions, then, for ceramics, having a dielectric nature and a high melting point, such effects are less pronounced. In this case, for ceramics, the effects of irradiation flux density can have a greater effect on structural changes and flaw formation at the same irradiation fluences, as well as atomic displacements.

## 2. Materials and Methods

Polycrystalline AlN ceramics stabilized by yttrium oxide (Y_2_O_3_) with a hexagonal type of crystal lattice such as wurtzite, spatial syngony of P63mc (186) and parameters a = 3.1149 Å, c = 4.9820 Å, and c/a = 1.599 were chosen as an object for research. The difference between the values of the crystal lattice parameters and the values of the standard (PDF-01-076-0702, a = 3.11 Å, c = 4.98 Å) is associated with deformation distortions that occur during the manufacture of ceramics by hot pressing.

The samples were irradiated at the DC-60 heavy ion accelerator located at the Institute of Nuclear Physics in Astana, Kazakhstan. Xe^23+^ heavy ions with energies of 230 MeV (1.75 MeV/nucleon) were chosen for irradiation. The irradiation fluence was 5 × 10^12^ ion/cm^2^, which, in terms of atomic displacements, is 0.0001 dpa along the entire ion path and 0.0004 dpa at the maximum path length, where nuclear losses of incident ions dominate. To test the hypothesis of the influence of the irradiation flux density on the degree of radiation damage and the consequences caused by them in the damaged layer at one irradiation fluence (and, as a result, one value of atomic displacements), heavy ion irradiation was carried out with the control of the beam current and its variation from 15 nA to 45 nA. The choice of conditions for the variation of the ion beam current density was limited by the capabilities of the DC-60 accelerator, for which the range of ion beam current density for Xe^23+^ heavy ions with energies of 230 MeV is 15–50 nA. At the same time, achieving the maximum value is difficult due to the possible occurrence of an uneven distribution of the beam, which will lead to an uneven set of fluence during irradiation. Variation of the ion beam current made it possible to vary the value of the ion flux density, which was from 3.2 × 10^8^ to 1.1 × 10^9^ ion/cm^2^·s. When the ion flux density is varied during irradiation, the time for which the required fluence value and, accordingly, the atomic displacements are reached decreases at high ion beam currents and increases with a decrease in the ion beam current. With such a variation, the radiation damage accumulation rate, or the so-called rate of atomic displacements (*R_d_*) changes, which can be calculated using Formula (1):

(1) $$R_{d}=\frac{N\times \wedge \times \sigma^{el}}{4E_{d}}\times E_{ion}\times \varphi$$

where $\wedge =\frac{4A}{{\left(1+A\right)}^{2}}$, *N*—to the number of target atoms per cc, *σ^el^*—the displacement cross-section, *E_d_*—the displacement threshold energy, *E_ion_*—the total ion energy, and *φ*—the ion flux. To derive this formula, calculations and assumptions made in the works [24,25,26] were used.

According to calculations, the value of *R_d_* for the selected irradiation conditions was 3.4 × 10^−8^ dpa/s, 6.6 × 10^−8^ dpa/s, and 8.7 × 10^−8^ dpa/s for ion beam currents of 15 nA, 30 nA, and 45 nA, respectively. From the presented calculation data, it can be concluded that an increase in the ion beam current from 15 nA to 45 nA leads to a more than a twofold increase in the rate of accumulation of atomic displacements, which makes it possible to reduce the time of sample irradiation. Moreover, an increase in the rate of the accumulation of atomic displacements can result in a change in the radiation damage evolution in the near-surface layer due to a change in the rate of their formation.

**Figure 1:**
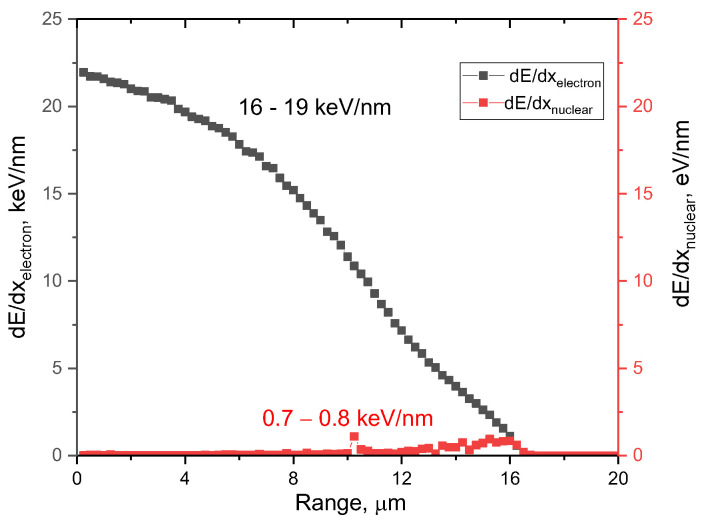
Simulation results of ionization losses of incident Xe^23+^ ions with an energy of 230 MeV.

The SRIM Pro 2013 software code was used to estimate the influence of the contributions from the ionization losses of incident ions during interaction with the electron and nuclear subsystems of the near-surface layer, as well as to determine the maximum range. Using the Kinchin–Pease model and considering the cascade interactions, the simulation was carried out. The sample density was chosen to be 3.26 g/cm^3^, and the displacement threshold energy (E_d_) was 19 eV and 55 eV for the N and Al atoms [27]. Figure 1 demonstrates the results of the simulation of the ionization losses of incident Xe^23+^ ions with an energy of 230 MeV (1.75 MeV/nucleon) in a 20-μm-thick target composed of polycrystalline AlN.

The overall view of the presented dependence of the change in the ionization losses of incident ions in the ceramic material indicates that, over most of the ion path in ceramics (approximately 10–12 μm), the main contribution to the defect formation processes is made by the interactions of incident Xe^23+^ ions with the electronic subsystem, which leads to ionization processes and the appearance of cascades of secondary knocked out electrons. At the same time, in the case of dielectric ceramics, ionization processes associated with a change in the electron density along the trajectory of Xe^23+^ ions in the ceramic material result in the appearance of anisotropic distortion and redistribution of electron density, which, due to the dielectric nature of ceramics, is not reversible. At the same time, the differences in the values of energy losses during the interaction of incident ions with the electronic and nuclear subsystems by more than two orders of magnitude indicate that, at the chosen irradiation fluence, the value of atomic displacements (associated with nuclear losses) does not make a significant contribution to structural distortions and amorphization processes (dpa value is not more than 0.0001 dpa). Moreover, the dominant role in the processes of deformation distortions of the crystal structure will be played by the electronic losses of ions, leading to the ionization of the damaged layer, as well as the formation of vacancy defects.

The study of structural features and their changes depending on the current density of the ion beam was studied using the X-ray diffraction method. X-ray diffraction patterns were taken using a D8 Advance ECO diffractometer (Bruker, Berlin, Germany). The diffraction patterns were obtained in the Bragg–Brentano geometry, the survey was carried out in the angular range of 2θ = 30–90°, with a step of 0.03°. Moreover, to evaluate the anisotropic distortion effects or texturing effects, the studied samples were studied using the method of obtaining X-ray diffraction patterns in the survey geometry of φ = 0–360°, consisting of obtaining a series of X-ray diffraction patterns taken in the Bragg–Brentano geometry with successive rotation of the sample relative to the perpendicular axis of the sample holder by φ = 10°. In this case, in the case of shooting a similar series of diffraction patterns, the fact of the same area of the X-ray diffraction of the sample when rotated by φ = 10° is considered. The resulting series of X-ray diffraction patterns in the geometry of φ = 0–360° is a set of 37 diffraction patterns reflecting the effects of texture and structural distortions associated with anisotropic changes in the deformation distortions of crystallites. The evaluation of the intensities of diffraction reflections, as well as changes in their contributions, was used to determine the effects of texturing, reflecting the influence of deformation distortions and residual mechanical stresses on the reorientation of crystallites in the damaged layer. The texture coefficients were analyzed according to the technique proposed in [28,29].

The optical properties were studied using UV–Vis spectroscopy and Raman spectroscopy. Transmission and absorption spectra were obtained using a Specord 250 plus (Analytic Jena, Upland, CA, USA). These spectra were obtained by taking samples in the wavelength range from 300 to 1000 nm, with a step of 0.1 nm, the analysis of which was carried out by comparing the data of the spectra of irradiated samples with the original spectrum of the unirradiated sample. The band gap (*E_g_*) was determined by analyzing the obtained UV–Vis spectra and Tauc’s plots using Formula (2):

(2) $$\alpha =A{\left(h\nu -E_{g}\right)}^{1/2}$$

where *A* is a constant and *hν* is the photon energy.

Raman spectra were obtained using a Nanofinder SP Raman microscope (Sol Instruments, Minsk, Belarus) using a single-mode longitudinal excitation laser with a wavelength of 532 nm (Torus, Laser Quantum, Bedford, MA, USA).

## 3. Results and Discussion

**Figure 2:**
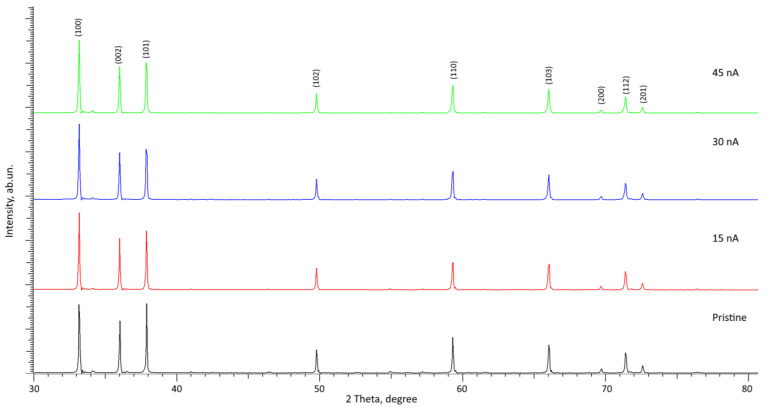
Results of X-ray diffraction of the studied ceramic samples depending on the ion beam current in comparison with the X-ray diffraction data of the sample in the initial state.

Figure 2 presents the results of a comparative analysis of changes in the diffraction patterns of the studied samples of AlN ceramics subjected to irradiation with Xe^22+^ heavy ions with an irradiation fluence of 5 × 10^12^ ion/cm^2^ with varying beam current. The comparison of X-ray diffraction patterns was performed with the obtained diffraction pattern of a non-irradiated ceramic sample. The overall view of the presented diffraction patterns, depending on the beam current, indicates the absence of any changes associated with the appearance of new diffraction peaks or the stratification of the observed reflections, which indicates the absence of polymorphic transformation processes in the irradiated layer, and the high resistance of ceramics to partial amorphization. Resistance to amorphization caused by irradiation is expressed in the absence of pronounced changes in the shape of diffraction reflections, as well as their appearance of asymmetry in them, which is characteristic of heavily deformed structures [30]. The main changes in the diffraction patterns of the irradiated samples in comparison with the diffraction pattern of the samples in the initial state are expressed in a slight shift of the diffraction maxima to the low-angle region 2θ⁰, indicating the deformation of the crystal structure due to irradiation, as well as a change in the ratio of intensities for the main most intense diffraction reflections (100), (002), and (101). Such a change in the intensity ratio indicates the effect of the reorientation of texture planes because of irradiation, which can be attributable to the effects of various degrees of deformation distortions, as well as the accumulated defective fraction in the damaged layer. In this case, the absence of the appearance of new diffraction reflections or the stratification of the observed reflections indicates the absence of processes of phase or polymorphic transformations in the composition of ceramics because of irradiation. It should be noted that in the case of AlN ceramics having a hexagonal type of crystal lattice, according to several works [11,31,32], irradiation with heavy ions does not cause the formation of structurally changed regions in the form of discontinuous or extended latent tracks, which are observed in silicon nitride. Several authors [33,34,35] attribute such differences in nitride ceramics to a higher binding energy in aluminum nitride, which prevents the formation of latent tracks. At the same time, it was shown in a few works [36,37,38] that the main effects of structural changes caused by exposure to ionizing radiation are associated with deformation distortions and stresses, which can result in the appearance of structurally disordered regions in the damaged layer of ceramics and which have an adverse impact on the properties of ceramics.

**Figure 3:**
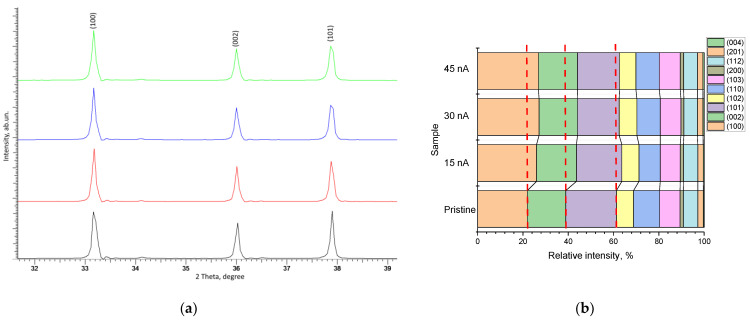
(**a**) X-ray diffraction patterns of the main diffraction reflections, showing alterations in the intensities and shapes of the main diffraction maxima (100), (002), and (101); (**b**) Results of the estimation of the contributions of the intensities of diffraction reflections, showing the effects of the reorientation of texture directions depending on the type of external action (dotted red lines indicate the position of the intensity contributions in the case of unirradiated ceramics).

Figure 3a demonstrates the dynamics of the three most pronounced diffraction reflections, (100), (002), and (101), the change in which indicates a structural distortion of the crystal lattice along the a and c axes, and the change in intensities indicates the texture reorientation processes depending on external influences. As can be seen from the data presented in Figure 3a, an increase in the ion beam current leads to the reorientation of the main diffraction reflections associated with a change in their intensity, as well as a distortion of the shape of the diffraction reflections, indicating a deformation distortion resulting from the accumulation of radiation damage in the form of point and vacancy defects. The shift of diffraction reflections to the region of small angles, as well as the distortion of their shape, which is clearly distinguishable for the reflections (100) and (101), indicate the appearance of a damaged layer in the structure associated with the formation of deformation tensile stresses caused by irradiation. In this case, the most pronounced observed changes in the shape of the (100) and (101) diffraction reflections indicate the possible occurrence of an anisotropic distortion of the crystal lattice along the a axis. The data presented in Figure 3b in the form of a diagram of changes in the contributions of the texture coefficients of the most pronounced diffraction reflections quite clearly reflect the effect of the ion beam current density on the reorientation of texture directions in comparison with the initial sample. In this case, it should be noted that an increase in the ion beam current density has the greatest effect on the (100) texture direction, which indicates reorientation processes as a result of ionization processes and the radiation damage formation.

As shown in [39,40], the measurement of X-ray diffraction patterns of the most pronounced diffraction maxima in the geometry of φ = 0–360° makes it possible to estimate the isotropy of structural deformations, which are expressed in changes in the intensity and angular position of reflections. At the same time, the observed differences in the intensities of diffraction reflections in the geometry φ = 0–360° indicate not only deformation distortions of the crystal lattice but also the occurrence of anisotropic spatial distortions in the damaged layer. Anisotropic distortion primarily arises during structure deformation, as well as the formation of disorder regions during the accumulation of point defects and their subsequent agglomeration. At the same time, in a few works [39,41], it was shown that structures with a hexagonal type of crystal lattice are most susceptible to anisotropic distortion.

**Figure 4:**
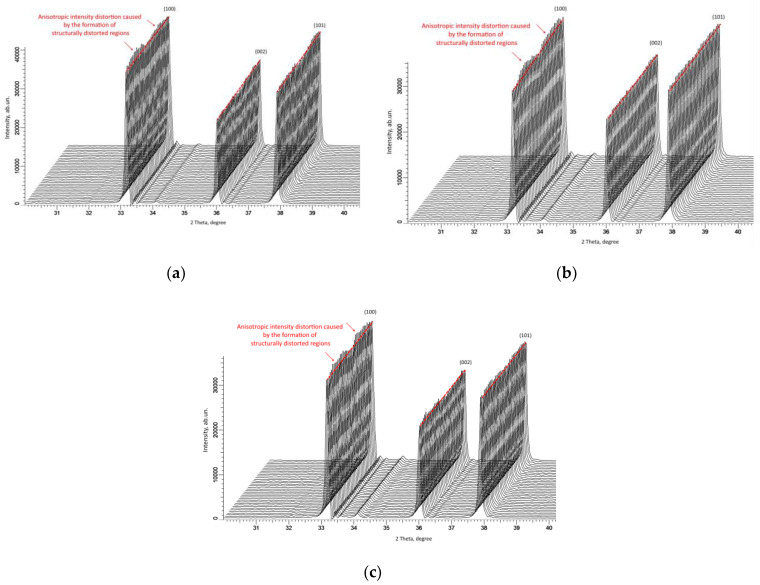
Results of estimating the alteration in the intensity of diffraction reflections (100), (002), and (101) in the survey geometry of φ = 0–360° for samples irradiated with different current densities: (**a**) 15nA; (**b**) 30 nA; and (**c**) 45 nA.

As can be seen from the presented data of X-ray diffraction patterns of samples irradiated with different ion beam currents, the observed changes in the angular dependence of the intensities of diffraction peaks are characteristic of the occurrence of anisotropic volumetric deformation in the structure, the appearance of which is associated with the formation of structurally altered areas caused by the passage of incident ions in the damaged layer (see data in Figure 4). The formation of such structurally distorted regions or inclusions in a damaged ceramic layer can be due to several factors. Firstly, during the interaction of heavy ions with the crystal structure of the target, the main mechanisms for transferring the kinetic energy of incident ions are associated with the processes of ionization and the subsequent transformation into thermal energy. At the same time, due to high binding energies (more than 40 eV [35]), the crystal structure of aluminum nitride can withstand large deformation distortions caused by irradiation. Secondly, the dominant process in the ionization losses of incident ions during irradiation with high-energy heavy ions is the interaction of ions with electron shells, which leads to the appearance of ionization effects and, therefore, the redistribution of the electron density in the dielectric matrix. The result of such interactions can be the appearance of an anisotropic distortion of the electron density, as well as the formation of vacancy defects of the V_Al_ or V_N_ type, the appearance of which can lead to additional structural distortions associated with their migration. Thirdly, the observed effects of changes in texture coefficients with variation in the ion beam current density indicate the appearance of the effects of the reorientation or distortion of the shape of crystallites in the structure of the damaged layer. When it comes to the assumption of a spherical shape of crystallites (regions of coherent X-ray scattering), the variation in the position of the sample during its X-ray photography should not affect the intensity of diffraction reflections. Moreover, in the case of a distorted shape of crystallites (regions of coherent scattering), the interaction of X-ray radiation with the crystal structure, depending on the position of the sample, will be different. In some cases, such effects are associated with the appearance of texture effects [42,43] under external mechanical influences. In the case of irradiation, as was shown in [44], the effects caused by irradiation with heavy ions are comparable to the effects of mechanical residual stresses in the damaged layer, the accumulation of which can lead to a destructive change in the properties of ceramics. Moreover, the emergence of residual mechanical stresses occurs because of the dominance of electronic ionization losses along the trajectory of ions in the material, leading to the formation of structurally changed areas in the damaged layer. It should also be noted that, in [44], a close to equiprobable distribution of residual mechanical stresses was shown almost along the entire ion motion trajectory, which, in turn, may indicate an isotropic change in material properties. At the same time, the observed anisotropic distortions of the shape and intensity of diffraction reflections during shooting in the geometry φ = 0–360°, considering the foregoing, can be attributable to the effects of overlapping structurally changed areas in the damaged layer. In a detailed analysis of anisotropic changes in the intensities of diffraction reflections with variation in the ion beam current density, it can be noted that an increase in the current density leads to a more pronounced anisotropy associated with the formation of local maxima and minimum. The appearance of such effects may be due to both anisotropic changes in structural properties and more pronounced texturing effects.

It should also be noted that a large difference between electronic and nuclear losses can lead to the formation of amorphous tracks or inclusions in the structure of ceramics, for silicon nitride (Si_3_N_4_), which was reported in [32,33,34]. However, for aluminum nitride (AlN) ceramics, such latent tracks were not observed, and several authors [35,45] associate the main structural changes caused by irradiation with the accumulation of residual mechanical stresses resulting from deformation distortions of the crystal lattice. The observed effects of changes in the intensities of diffraction reflections with variations in the ion beam current density also indicate the effect of the initialization of processes associated with the recrystallization or reorientation of grains as a result of the accumulation of deformation distortions and stresses. Moreover, it is clearly seen, from the data presented, that this effect is most pronounced with an increase in the ion beam current density, when the damage accumulation rate becomes higher at the same accumulated fluence. From this, it can be concluded that the current density of the ion beam and, therefore, the rate of accumulation of radiation damage plays an important role in the structural changes in ceramics caused by irradiation.

In the case of the analysis of X-ray data obtained using the X-ray diffraction method, the changes indicate the presence of the effect of structural distortions associated with both mechanical residual stresses due to the interaction of incident ions with the crystal structure and the effects of anisotropic textural reorientation of crystallites. At the same time, X-ray diffraction data make it possible to evaluate volumetric structural changes; however, to determine the types of defects or to estimate the concentration of vacancy defects, optical or Raman methods should be used.

**Figure 5:**
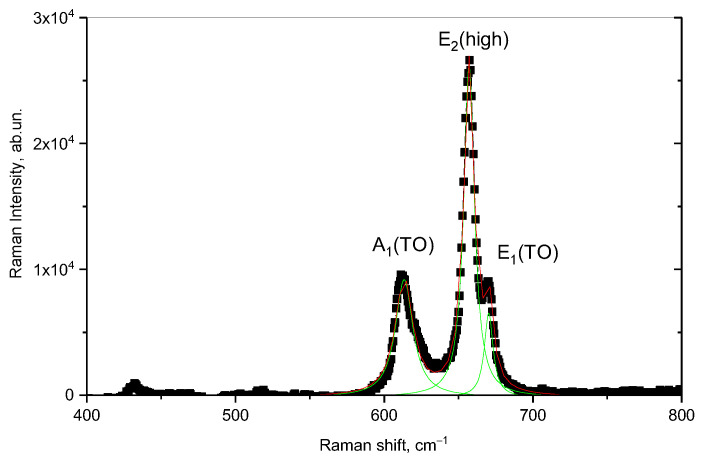
Raman spectrum of AlN ceramics in the initial state.

Figure 5 demonstrates the results of Raman spectroscopy measurements of the initial sample of AlN ceramics.

Approximation of the obtained spectral lines using the Lorentz functions made it possible to establish the presence of three main spectral lines in the range of 400–800 cm^–2^, characteristic of the three modes A_1_(TO), E_2_(high), and E_1_(TO) with maxima at 613, 657, and 670 cm^–2^, respectively. A slight deviation from the values of the maxima of the spectral lines presented in [46] is associated with the presence of defects in the structure of ceramics that arise during their manufacture. At the same time, in this work, the authors associate the intensity of the E_2_(high) spectral line with the vibrations of Al and N atoms, and its change in the case of broadening can be attributable to the effects of the substitution of N atoms by O atoms, as well as the formation of oxygen substitution vacancies for nitrogen (*O_N_*) or the formation of *ON–VAl* complexes. The presence of oxygen in the structure of ceramics can be due to the methodology for obtaining aluminum nitride ceramics from aluminum oxide powders, as well as the presence of stabilizing additives in the form of yttrium oxide (Y_2_O_3_), which is used to stabilize and strengthen ceramics. It should also be noted that the content of the stabilizing additive Y_2_O_3_ is no more than 1–2% (according to X-ray phase analysis), and the distribution of this additive in the structure is isotropic throughout the volume.

In the case of external influences on ceramic samples, the main changes will manifest themselves in the form of changes in the shape of spectral lines associated with their broadening (the FWHM value variation), as well as the shift of maxima, reflecting the formation and subsequent accumulation of residual mechanical stresses, leading to structure deformation. At the same time, the assessment of the change in the main spectral lines as a result of external influences makes it possible to assess the types of defects formed in the structure.

**Figure 6:**
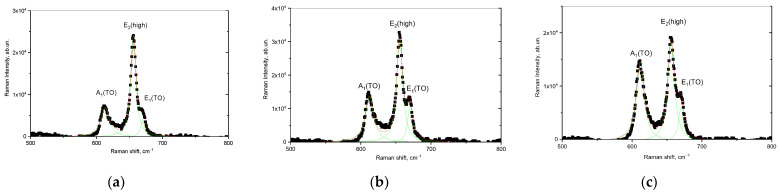
Raman spectra of AlN ceramics after irradiation with Xe^23+^ heavy ions with variation of the ion beam current: (**a**) 15nA; (**b**) 30 nA; and (**c**) 45 nA.

Figure 6 demonstrates the results of the Raman spectra of the studied AlN ceramics irradiated with Xe^23+^ heavy ions with different ion beam currents. The overall view of the presented Raman spectra is similar to the spectrum of non-irradiated ceramics; however, in the case of variation of the ion beam current, a change in the intensities of the spectral lines is observed, as well as from the half-widths (FWHM).

The deformation nature of the structural changes caused by irradiation is evidenced by the change in the shift of spectral lines (Raman shift). Moreover, as is known, the spectral line E_2_(high) is most sensitive to deformation distortions of the structure because of external influences [47]. At the same time, the analysis of the observed changes in the spectral lines showed variations in the current density of the ion beam and that there are two types of characteristic changes in the Raman spectra. The first type of changes is associated with a change in the intensity and magnitude of the FWHM of the spectral lines, which indicates the effect of structural disorder and the amorphization of the structure. When it comes to irradiation with heavy ions under high-dose irradiation, the decrease in the intensity of spectral lines and their broadening with increasing FWHM indicates the effect of amorphization and the destructive disordering of the crystal structure of samples subjected to irradiation [44].

The second type of changes is associated with the shift of the spectral lines relative to the maximum position of the spectral lines characteristic of the original sample. These displacements characterize the presence of residual mechanical stresses arising because of deformation distortions during the interaction of incident ions with the ceramic crystal structure, and the “−” or “+” sign when calculating this value characterizes the type of these stresses—compressive or tensile.

An assessment of the residual mechanical stresses arising in the structure of the near-surface layer of ceramics, as well as the displacement of spectral lines, is presented in Table 1. The assessment of deformation distortions associated with residual mechanical stresses (*σ_xx_*) was carried out using Formula (3), where the value of the deformation coefficient is *k* = 3.7 cm^−1^/GPa, and ∆ω is the Raman shift of the irradiated sample compared to the initial value.

∆*ω* = *kσ_xx_* (3)

As can be seen from the data presented in Table 1, a change in the irradiation conditions (an increase in the ion beam current density) leads to a shift and broadening of the Raman reflections, indicating the processes of deformation distortion, as well as the appearance of structurally disordered regions in the damaged layer.

**Figure 7:**
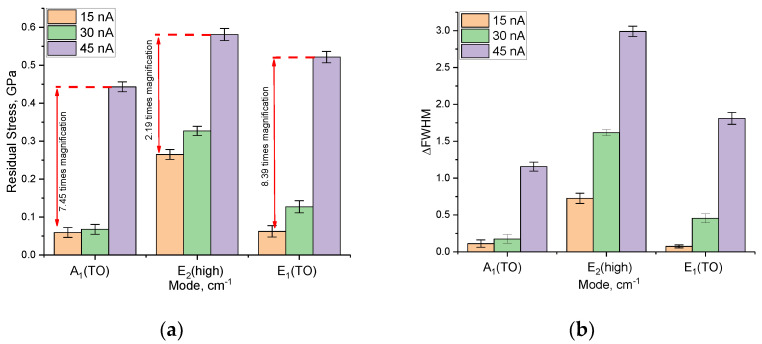
(**a**) Results of the assessment of the change in the value of residual mechanical stresses in the damaged layer of ceramics, determined using Raman spectroscopy, in the case of different irradiation conditions; (**b**) Results of changes in the FWHM value of spectral lines under different irradiation conditions.

Figure 7a demonstrates the results of the assessment of the change in the value of residual mechanical stresses *σ_xx_* for three spectral lines A_1_(TO), E_2_(high), and E_1_(TO), depending on the type of external action. The overall view of the presented data on the change in the magnitude of residual stresses indicates a significant effect of the variation in the ion beam current density on the shift in the positions of the maxima of the spectral lines, as well as the formation of residual mechanical stresses in the structure. At the same time, the nature of changes in the magnitude of residual stresses indicates that these stresses are tensile stresses characterized by deformation distortions of the structure, leading to an increase in its volume and the distortion of crystalline and chemical bonds.

The variation in the FWHM value of the spectral lines, indicating the formation of structurally disordered inclusions in the damaged layer, is shown in Figure 7b. The overall appearance of the observed changes in the FWHM value indicates that the processes characteristic of the formation of structurally disordered regions in the damaged layer are most pronounced at high ion beam current densities, which, in turn, results in an acceleration of the accumulation of amorphous inclusions in the damaged layer. In the case of low current densities of the ion beam, the alterations in the ∆FWHM value are most pronounced only for the E_2_(high) spectral line, the change in which, as shown in [47], is most sensitive to deformation distortions, as well as to the accumulation of amorphous inclusions.

As can be seen from the data presented, the most pronounced changes in the spectral lines upon irradiation are observed for the E_2_(high) mode, which, as mentioned above, characterizes the change in the vibrations of the Al and N atoms, as well as the formation of *ON–VAl* complexes. Moreover, the variation of the ion beam current density from 15 to 45 nA leads to pronounced changes in the shifts of the spectral lines, which indicates a more intense formation of defective inclusions at the same irradiation fluence. In turn, small changes in the magnitude of residual mechanical stresses at an ion beam current density of 15 and 30 nA (the difference is less than 5–10%) indicate that at low ion beam densities, the rate of accumulation of radiation damage and the consequences caused by their formation is much lower than at high densities. This effect can be explained by the fact that at high current densities of the ion beam, there are effects of the cascade mixing of defects in the structure of the damaged layer, associated with an increase in the concentration of the resulting *ON–Val* vacancy complexes, the appearance of which leads to the partial amorphization of the structure. In this case, a high concentration of accumulated residual mechanical stresses can lead to a destructive change in the properties of the damaged layer, as well as the formation of metastable deformed inclusions in it, the presence of which will lead to a decrease in the stability of ceramics.

Analyzing the data presented, we can conclude the following. The variation of the ion beam current density at the same irradiation fluence leads to different rates of accumulation of radiation damage associated with the formation of many *ON–VAl* vacancy complexes, a rise in the concentration of which results into partial amorphization of the damaged layer. Moreover, in the case of low current densities of the ion beam, the dominant role in structural changes is played by deformation distortions associated with the accumulation of residual mechanical stresses in the damaged layer, while amorphization processes are less pronounced.

Another of the most important methods for the non-destructive testing of structural changes and their influence on the optical and electronic properties of ceramics is the method of evaluating optical spectra in the UV–Vis range. Changes in optical characteristics, such as the transmission, absorption, shift of the fundamental absorption edge, make it possible to evaluate the effect of radiation-induced damage caused by irradiation on the properties of ceramics.

**Figure 8:**
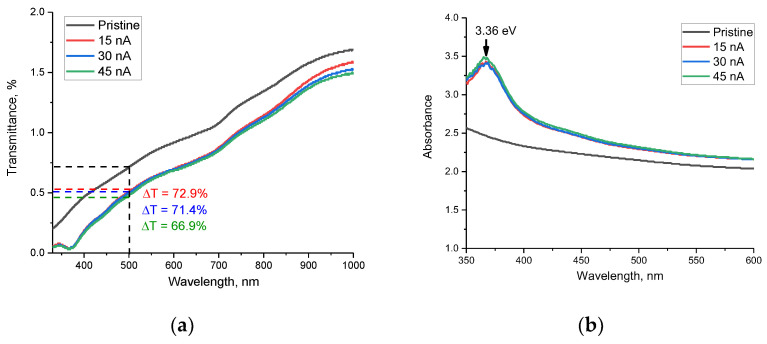
Optical UV–Vis spectra of AlN ceramics irradiated with Xe^23+^ heavy ions: (**a**) Transmittance; (**b**) Absorbance.

Figure 8 shows the optical transmission spectra for AlN ceramic samples taken in the range of 330–1000 nm. The spectra obtained can be characterized by the presence of a fundamental absorption edge in the short-wavelength region, as well as by the presence of a multiphonon spectrum of lattice vibrations in the long-wavelength region (above 700 nm). As can be seen from the presented data, the main changes observed for the irradiated samples are characterized by a shift in the fundamental absorption edge, as well as a decrease in the transparency of the spectra in the entire measured wavelength range. It is also worth mentioning that the variation of the ion beam current with its increase leads to a decrease in transparency, which is most pronounced in the near-IR region (800–1000 nm). Such alteration in the transmission spectra in the region of 800–1000 nm can be due to the difference in the concentration of the defective fraction, which leads to changes in the multiphonon spectra of lattice vibrations due to the formation of additional defective and distorting inclusions. The decline in the transparency of ceramics, as shown in [48], is primarily attributable to the appearance of additional grain boundaries in the structure, which are potential scattering centers, a change in the concentration of structural defects located near the grain boundaries and the deformation distortions of the structure. It should be noted that the established formation of *ON–VAl* vacancy complexes also affects the transmission efficiency due to the formation of additional absorbing centers. In the case of irradiation, all three factors can influence reducing the transmission capacity and increasing the refractive index. Moreover, the decrease in transmission associated with a decrease in the transparency of ceramics can be due to the formation of oxygen-containing inclusions in the structure. The presence of these inclusions in the structure of ceramics leads to a decrease in transparency, as well as a shift in the fundamental absorption edge. At the same time, the analysis of the obtained optical transmission spectra depending on the ion beam current revealed that the decline in transparency is most pronounced at an ion beam current of 45 nA, and the decrease in optical transparency is more than 33%, while for samples irradiated with ions with a current of 15 nA and 30 nA, the decrease in transparency is less than 28% and 29%, respectively.

When analyzing the absorption spectra presented in Figure 8b, two types of changes were found associated with changes in the irradiation conditions of ceramics. A common type of change for all irradiation conditions, with variation in the ion beam current, was the formation of a broad spectral absorption band in the region of 350–400 nm, with a maximum at 365–366 nm, which corresponds to a photon energy of 3.36 eV. In this case, a change in the irradiation conditions with an increase in the ion beam current leads to an increase in the intensity of this spectral band, which indicates a change in the concentration of absorbing centers in ceramics. The presence of this spectral band, according to [49], is associated with the formation and subsequent recombination of donor–acceptor pairs between V_Al_ vacancies and oxygen atoms replacing nitrogen atoms. At the same time, a growth in the intensity of this band indicates a variation in the concentration of vacancy defects, and a slight shift of the maximum may be attributable to deformation distortions in the structure that arise during the radiation damage accumulation.

**Figure 9:**
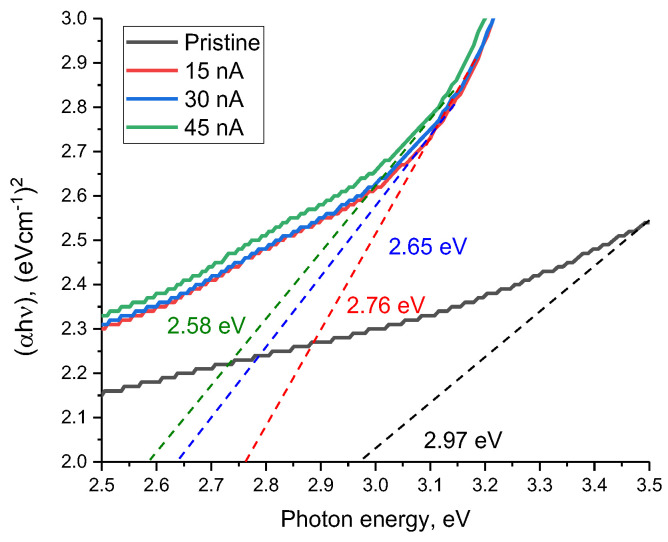
Results of estimation of the shift of the fundamental absorption edge in AlN ceramics upon irradiation with Xe^23+^ ions with different ion beam currents (dotted lines indicate the tangents to the absorption curves, according to the constructions of Tauc. The values shown in the figure were determined using Formula (2)).

Figure 9 reveals the results of estimating the change in the band gap (E_g_) depending on the ion beam current, reflecting the shift of the fundamental absorption edge, as well as the change in the electron density distribution in the damaged layer.

As can be seen from the presented data, irradiation with heavy Xe^23+^ ions results in a shift of the fundamental absorption edge to the long wavelength region, which indicates a change in the band gap between the valence band and the conduction band, as well as, in the case of dielectric ceramics, the appearance of anisotropy in the distribution of electron density in the damaged layer. Moreover, these changes are most pronounced in comparison with the initial value of the shift of the fundamental absorption edge in the case of irradiation with an ion beam current density of 45 nA. It should also be noted that an increase in the ion beam current density from 30 to 45 nA leads to less pronounced changes in the shift of the fundamental absorption edge than with an increase in the density from 15 to 30 nA. Such a difference can be attributable to the effects of partial saturation of changes in the electron density in the damaged layer, as well as the appearance of anisotropy in the distribution of electrons in the damaged layer. The formation of an additional induced absorption band at 3.36 eV in the composition of the damaged layer has a good correlation with the formation of *ON–VAl* vacancy complexes, which lead to an increase in absorption and a change in the band gap.

**Figure 10:**
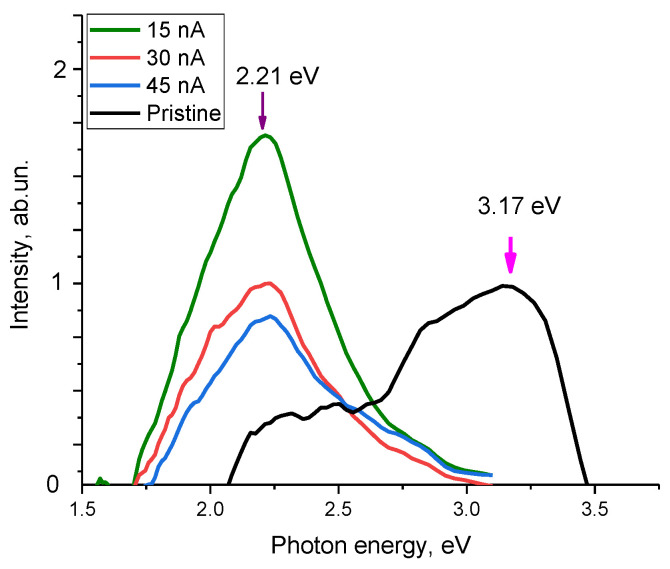
Photoluminescence spectra of AlN ceramics before and after heavy ion irradiation with varying ion beam current density (to represent the presence of luminescence spectra, the background was subtracted to reflect the presence of spectral maxima and changes in their intensity with varying irradiation conditions).

Figure 10 demonstrates the optical photoluminescence spectra of ceramics as a function of the ion beam current density, reflecting changes in the optical properties of ceramics.

The overall form of the observed changes indicates the formation of luminescence centers in the structure of the irradiated samples, the concentration of which varies depending on the irradiation conditions. In the case of the initial sample, the resulting spectrum can be characterized by two Gaussian distributions with local maxima at 3.17 eV and 2.2 eV. The spectral band at 2.2–2.5 eV is characteristic of the presence of oxygen-vacancy complexes of the *ON–VAl* type, the content of which is insignificant in the case of the initial samples. The band with a maximum at 3.17 eV may be due to the recombination of donor-acceptor pairs associated with *V_Al_* vacancies, the presence of which may be due to ceramic fabrication processes [50]. In the case of irradiated samples, regardless of the variation in the ion beam current density, the dominant spectral band is 2.2–2.5 eV, the intensity of which increases with the current density. At the same time, a rise in the ion beam current density from 30 to 45 nA results in to an almost twofold increase in the intensity of the spectral band characteristic of vacancy complexes of the *ON–VAl* type, which indicates a higher defect formation rate in the damaged layer compared to lower current densities. In turn, the dominance of this band indicates an increase in the concentration of defects of the *ON–VAl* type, and the asymmetric shape of the spectral line in the region of 2.5–2.7 eV at an ion beam current density of 45 nA can be due to the formation of recombination processes involving nitrogen vacancies (*V_N_*), the presence of which is associated with deformation distortions of the crystal structure, as well as an increase in the contribution of structurally disordered inclusions in the damaged layer.

Thus, summing up the obtained data on changes in optical properties, we can draw the following conclusions. The dominant type of structural changes caused by irradiation at current densities of 15–30 nA is the formation of vacancy complexes of the *ON–VAl* type. The formation of these complexes is most characteristic of structural changes in aluminum nitride ceramics associated with external influences [41,48,49,50]. Moreover, as shown in [41], the formation of vacancy complexes of the *ON–VAl* type is the most preferable defect formation mechanism for nitride ceramics, while the presence of vacancies of the *V_Al_* type in the structure of irradiated ceramics can also occur, but their concentration is much lower than the concentration of *ON–VAl* vacancy complexes. The formation of *ON–VAl* vacancy complexes occurs as a result of the higher mobility of oxygen in the structure of ceramics, the presence of which is due to stabilizing additives in the composition of ceramics (Y_2_O_3_). At the same time, the formation of these *ON–VAl* complexes leads to a sharp increase in absorption, which is observed both in the case of the analysis of optical spectra and luminescence spectra.

While irradiation with heavy ions with a current density of 45 nA, in addition to *O_N_–V_Al_*, the formation of V_N_ vacancies is observed, the presence of which may be due to the deformation distortion of crystalline and chemical bonds, leading to the formation of vacancies because of their rupture. An alteration in the fundamental absorption edge value, as well as a decrease in the transmission of ceramics, indicates a change in the electron density in the damaged layer, associated with the anisotropic distortion of the crystal structure and texture effects, leading to the appearance of additional absorbing and refractive centers in ceramics.

## 4. Conclusions

During our studies, it was found that the variation of the ion beam current density during the irradiation of ceramics leads to various radiation damage accumulation mechanisms associated with the formation of *ON*–*VAl* vacancy complexes, as well as disordering effects caused by the accumulation of residual mechanical stresses.

During the analysis of the Raman spectra of irradiated ceramics, it was revealed that at low current densities (15–30 nA), the dominant process in the mechanisms of defect formation is the formation of *ON*–*VAl* vacancy complexes and deformation distortions, while at an ion beam current density of 45 nA, an increase in the concentration of structurally disordered regions leading to partial amorphization is observed in the structure of the damaged layer.

Analyzing the results of the study of structural and optical changes in the properties of AlN ceramics with varying ion beam current density, we can conclude that, in the case of using heavy ions to simulate radiation damage comparable to reactor exposure, one should consider not only the type of impact, but also the rate of formation and accumulation of radiation damage. In the case of low current densities of the ion beam, structural changes are mainly associated with the formation of vacancy and point defects, which can result in the formation of tensile-type deformation distortions. At the same time, at high ion beam current densities, these effects are accompanied by the partial amorphization of the damaged layer due to an increase in structurally disordered regions, the appearance of which is due to the high density of deformation distortions and residual mechanical stresses, as well as the formation of *V_N_*-type vacancies.

## Figures and Tables

**Table 1:** Data on the position of the maximum and the FWHM value of the spectral lines of the studied ceramics depending on the type of exposure.

**Raman Shift**	**Raman Shift, cm^−1^**			
	**Pristine**	**15 nA**	**30 nA**	**45 nA**
A_1_(TO)	613.41	613.19	613.16	611.77
E_2_(high)	657.06	656.08	655.85	654.91
E_1_(TO)	670.28	670.05	669.81	668.35
**FWHM**	**FWHM, cm^−1^**			
	**Pristine**	**15 nA**	**30 nA**	**45 nA**
A_1_(TO)	14.34	14.45	14.52	15.50
E_2_(high)	8.62	9.35	10.24	11.61
E_1_(TO)	7.16	7.23	7.61	8.97

## Data Availability

Not applicable.
